# Advancements in Hydrogel Application for Ischemic Stroke Therapy

**DOI:** 10.3390/gels8120777

**Published:** 2022-11-28

**Authors:** Ying Bai, Bing Han, Yi Zhang, Yuan Zhang, Yang Cai, Ling Shen, Yanpeng Jia

**Affiliations:** Department of Pharmacology, School of Medicine, Southeast University, Nanjing 210009, China

**Keywords:** ischemic stroke, hydrogel, biomaterials, drug delivery, regeneration

## Abstract

Ischemic stroke is a major cause of death and disability worldwide. There is almost no effective treatment for this disease. Therefore, developing effective treatment for ischemic stroke is urgently needed. Efficient delivery of therapeutic drugs to ischemic sites remained a great challenge for improved treatment of strokes. In recent years, hydrogel-based strategies have been widely investigated for new and improved therapies. They have the advantage of delivering therapeutics in a controlled manner to the poststroke sites, aiming to enhance the intrinsic repair and regeneration. In this review, we discuss the pathophysiology of stroke and the development of injectable hydrogels in the application of both stroke treatment and neural tissue engineering. We also discuss the prospect and the challenges of hydrogels in the treatment of ischemic strokes.

## 1. Introduction

**Figure 1:**
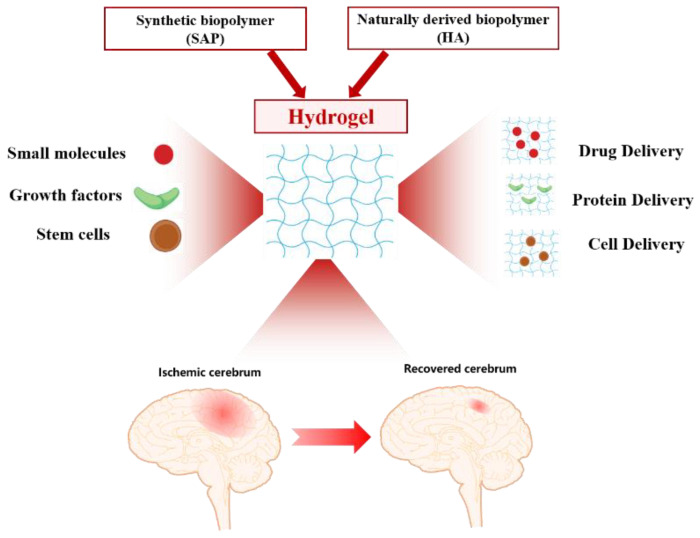
Schematic description of the hydrogel application in the treatment of ischemic strokes.

Strokes are among the significant causes of death and disability, affecting nearly 800,000 individuals annually [1,2]. A stroke is defined as an acute local blood flow condition in the central nervous system (CNS) caused by cerebral blood vessels. According to the cause, strokes are clinically classified into the following types: ischemic strokes and hemorrhagic strokes [3]. Approximately 85% of all acute strokes are ischemic strokes, which result from blockage of the blood supply to the brain. The remaining 15% of strokes involve hemorrhages [4]. This review mainly focuses on discussing ischemic stroke. When ischemia occurs, a lack of blood flow in the brain initiates a cascade of pathological processes, eventually leading to the death of brain cells and neurological damage [5]. Current treatment strategies for ischemic stroke focus on restoring blood flow and oxygenation in the damaged area through the administration of reperfusion medications (tissue plasminogen activator) or by mechanical means (thrombectomy) to reduce lasting damage and prevent the injury from further developing [6]. However, due to the narrow therapeutic time windows of tissue plasminogen activator administration (less than 4 h) and thrombectomy (less than 6 h), over 90% of patients cannot benefit from these treatments [7]. The disease usually progresses into a chronic phase, with life-long disability and failure of complete recovery. Due to their poor prognosis, strokes cause a major negative impact on the quality of life of patients and their families [8]. Therefore, due to the substantial burden on disabled stroke survivors and the complete lack of effective treatments that improve recovery, there is an opportunity to develop new strategies that enhance post-ischemic brain repair. Novel neuroprotectants have been designed and developed to treat ischemic strokes, but none of them have been approved clinically [9,10]. As is well known, to achieve an effective stroke therapy, various therapeutics must be efficiently delivered to the site of the ischemic brain. The significant hurdle that complicates the effect of stroke treatments is the blood-brain barrier (BBB) [11,12]. As a biological barrier that protects the brain from toxins and infections, the BBB also prevents most therapeutic drugs from being effectively delivered into the brain. Another reason why therapy fails is that therapeutic drugs involve problems such as short circulation times, poor stability and toxicity [13]. Therefore, to improve stroke treatments, novel methods must be developed to raise drug dosages within the injured brain. During the past decade, biomaterials have been widely applied to develop novel strategies for stroke treatments [14,15,16]. Among the various types of biomaterials, hydrogels represent the most extensively studied because of their unique chemical and mechanical properties. In recent years, hydrogels have emerged as a promising platform to enhance drug delivery locally for disease therapy since they are tunable to the tissue type for site-specific delivery [17,18]. As shown in Figure 1, in ischemic stroke therapy, hydrogels can be used as a vehicle for small-molecular drugs, large-molecular proteins, cells, and regenerating damaged brain tissue after a stroke [19,20]. Notably, hydrogels exhibit several advantages, including bypassing the blood-brain barrier and releasing therapeutic payloads locally. This review mainly focuses on the pathology of strokes and the development of hydrogel-based therapy for treating strokes.

### 1.1. Pathophysiology of Ischemic Strokes

Acute ischemic strokes occur due to a brain ischemia that results from thrombosis of a cerebral blood vessel [7,21]. An AIS is a complex polygenic and multifactorial disease with a close relationship to genetic factors and environmental factors [22,23]. Several studies have indicated that brain damage affects cells and causes cell death and dysfunction in brain tissue [23,24]. An AIS causes obvious oxygen and glucose deficiency and ionic gradient disturbance in neurons and other kinds of brain cells [25]. Then, multiple biochemical, physiological and molecular mechanisms are induced, which finally result in the alteration of neuron functions and extensive cell death in the ischemic core [26]. Dying cells in the brain can release dangerous signals that stimulate poststroke inflammation [26,27,28]. Damage-associated molecular patterns (DAMPs) promote the expression of chemokines and proinflammatory cytokines by resident immune cells, which help peripheral immune cells infiltrate the brain [29,30]. Moreover, these proinflammatory mediators cause the BBB to leak. After that, inflammation intensifies, and multiple cytokines are discharged in the damaged brain [31]. Cytokines represent key mediators during the immunoinflammatory reaction and are involved in the progression of cerebral infarction [32,33,34]. Pathologically related molecules could serve as potential targets that may help provide treatment methods that are urgently needed.

### 1.2. Limitations of Current Therapies

Appropriately treating acute ischemic stroke is necessary to reduce morbidity and mortality [35]. A large number of reports involve neuroprotective agents that were designed to block key steps during neuronal ischemia; however, almost no drug has been found to improve clinical outcomes. Currently, stroke therapies are mainly aimed at providing immediate reperfusion through thrombolytic mechanical recanalization of the obstructed blood vessels. Recombinant tissue plasminogen activator (rt-PA) is currently the only FDA-approved therapy [36]. In addition, the systemic delivery of candidate neural repair drugs is limited by the BBB and off-target effects; thus, promising candidate therapies for neural repair with potential clinical application value are limited [37]. For example, delivering promising therapeutic growth factors to the brain is very difficult [38]. Conventional methods of systemic delivery, such as intravenous administration, are relatively convenient and minimally invasive, but most growth factors fail to penetrate the BBB efficiently after tail vein injection. Therefore, high doses are necessary for the desired therapeutic result, which could lead to systemic side effects [39]. Combination methods, such as systemic administration, together with localized disruption of the BBB, are attractive strategies; however, opening the BBB non-selectively could cause toxic substances to enter the brain [40,41]. Local delivery approaches, such as intracerebroventricular infusion, could successfully bypass the BBB; however, invasive surgeries that damage the host are necessary [42].

## 2. Hydrogels for Regeneration and Recovery

Generally, the application of biomaterials in ischemic strokes is aimed at either delivering therapeutic drugs or functioning as a foreign extracellular matrix suitable for brain tissue growth. To date, hydrogels, as an important kind of biomaterial, have been commonly applied for cell delivery and drug delivery. Therefore, hydrogels may represent a potential resource that could be utilized to study biological changes after strokes and explore novel methods of therapy. In situ injectable hydrogels have been widely reported as a promising type of drug delivery system in various disease therapies, such as stroke therapy. A great advantage of hydrogels is that drug release is sustained and even controlled after a single injection.

### 2.1. Hyaluronan-Based Hydrogels

**Figure 2:**
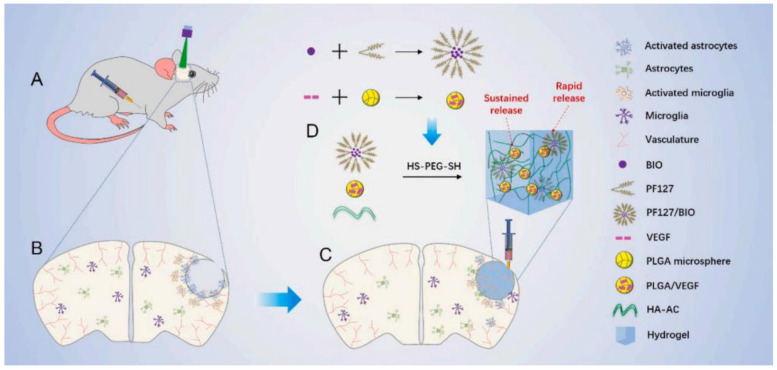
Schematic illustration of the applications of injectable hydrogels in the treatment of ischemic strokes in mice. (**A**,**B**) PT model in rats, in the PT model, blood flow disruption, microglia activation and astrocytes activation all occurred. (**C**) Injection of a dual-functional hydrogel into the infarct area (**D**) The preparation process of dual-functional hydrogel. Reproduced with permission from [56]. Copyright 2022, Elsevier.

**Figure 3:**
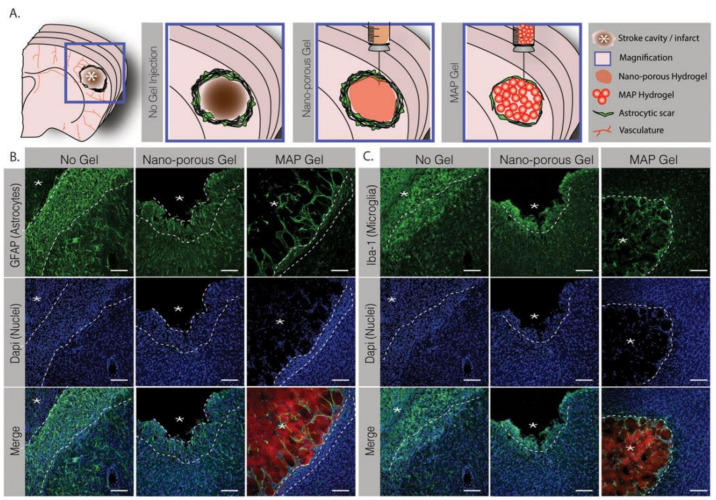
(**A**) Schematic illustration of a coronal mouse brain section, the magnified schematics show the No Gel, nanoporous and MAP hydrogel injection conditions. (**B**,**C**) Fluorescent images of GFAP and IBA-1 staining showing poststroke astrocytic and microglial response (scale bar: 100 μm), “*” represents stroke cavity as illustrated in (**A**). Reproduced with permission from [57]. Copyright 2011, Elsevier.

Hyaluronan (HA) can relieve the inflammatory response and further enhance cell survival via a CD44-mediated mechanism, which makes it a popular material for applications in drug delivery systems. As a popular biomaterial, HA shows excellent biocompatibility and biodegradability and is thus widely applied to treat ischemic strokes. HA is usually used together with methylcellulose (MC), called HAMC, as a hydrogel vehicle to deliver cortically specified neuroepithelial progenitor cells (cNEPs) into the stroke-injured brain. It has been well demonstrated that HAMC can significantly increase cell survival and distribution in several animal models of CNS disease [43,44,45]. Growth factor delivery is a promising therapeutic strategy for treating ischemic stroke. BDNF promoted functional recovery in several poststroke animal models [46]. However, the amount of BDNF that penetrates the BBB to the brain is too small to effectively treat strokes. Therefore, systemic injection of BDNF is not a recommended strategy. In addition, BDNF shows a short distribution time in brain tissue and is prone to degradation. It has been reported previously that delivering a single dose of BDNF to release sustainably from the stroke cavity could promote behavioral recovery in a mouse model [47]. An alternative method is to carry BDNF with a depot that was previously injected into the stroke cavity and release BDNF into the adjacent surrounding area. The infarct cavity is positioned in the center of the stroke and can be targeted for BDNF release [48]. In another study, the therapeutic effects of different doses of BDNF were tested by delivering them with a commercial hydrogel. Before implantation, the hydrogel was fully gelated to be delivered to the ischemic site. Due to elasticity, the hydrogel could be well conformed to the cavity and prevent the collapse of surrounding tissue [49]. A brain-compatible hyaluronan (HA) hydrogel loaded with BDNF was prepared and achieved sustained BDNF release over weeks. This system improved motor recovery, promoted the migration of immature neurons to the peri-infarct cortex and, finally, increased the survival rate of these cells. HA modification was also reported to increase the functions of the vehicle. For example, after being modified with an anti-Nogo-66 receptor antibody, HA hydrogel could promote regeneration more effectively in stroke therapy. This synthesized HA gel could serve as a scaffold for neural regeneration and support neural cell attachment, as well as deliver antibodies for sustained release [50]. Interestingly, the authors further developed a delivery system based on HA hydrogels loaded with both BDNF and VEGF-loaded PLGA microspheres to achieve controlled release of these two growth factors. In addition, hydrogel scaffolds could support the survival of neural stem cells in brain tissue [51]. Furthermore, HA hydrogel functionalized with an anti-Nogo receptor antibody and PLL was designed and prepared and loaded with PLGA microspheres encapsulating VEGF and Ang1. By implanting this composite system into the ischemic site, angiogenesis in situ was observed clearly when it was estimated in the MCAO model after implantation therapy. The results implied that this novel HA–PLGA hydrogel composite is a promising candidate for neural regeneration after a stroke [52]. Similarly, EGF and EPO can penetrate through the ischemic cortex when delivered epicortically from HAMCs [53]. Notably, protein release tends to occur rapidly from a hydrogel scaffold. For brain repair, a sustained release of two weeks was necessary. Therefore, it is difficult to achieve sustained release when loading protein into a hydrogel alone [54]. Considering these problems, Molly S. Shoichet et al. developed a new method for the sustained and local release of both EGF and EPO to the brain with temporal control properties. This delivery vehicle comprised HAMC hydrogel and polymeric particles containing EGF-PEG and EPO. Their study achieved a sequential release of EGF-PEG and EPO and triggered endogenous NSPCs in a mouse model, and obvious recovery was observed after strokes [55]. Wang et al. studied the sequential delivery of both the anti-inflammatory small-molecular drug BIO and the proangiogenic agent VEGF, utilizing a modified HA hydrogel to achieve a combination therapy effect for brain repair in ischemic strokes. As shown in Figure 2, the authors developed an HA hydrogel local delivery system in which both PF127/BIO NPs and PLGA/VEGF porous microspheres were loaded for sequential delivery. In this novel sequential delivery HA hydrogel system, PF127/BIO nanoparticles were released from the system more quickly due to their smaller particle size. The released PF127/BIO nanoparticles effectively decreased the inflammatory response after an ischemic stroke. Afterwards, the PLGA/VEGF porous microspheres with a larger diameter were released more slowly and more sustainably, which then enhanced angiogenesis in the infarct area. Overall, relieved inflammation and improved vascular regeneration promoted long-term neurological repair after a stroke [56]. Dr. Darling et al. prepared HA microporous annealed particles for brain repair processes after a stroke. For example, as shown in Figure 3, they utilized HA with FXIIIa for scaffold fabrication to improve the repair results. In contrast to the nonporous gels, HA microporous annealed particles could effectively enhance endogenous neural progenitor cell recruitment and vascular regeneration in the peri-infarct area [57]. In another work, they injected porous HA-Tet MAP scaffolds into the ischemic stroke mouse brain, which was proven to be biocompatible with a reduced inflammatory response and astrogliosis [58].

### 2.2. Other Naturally Derived Hydrogels

**Figure 4:**
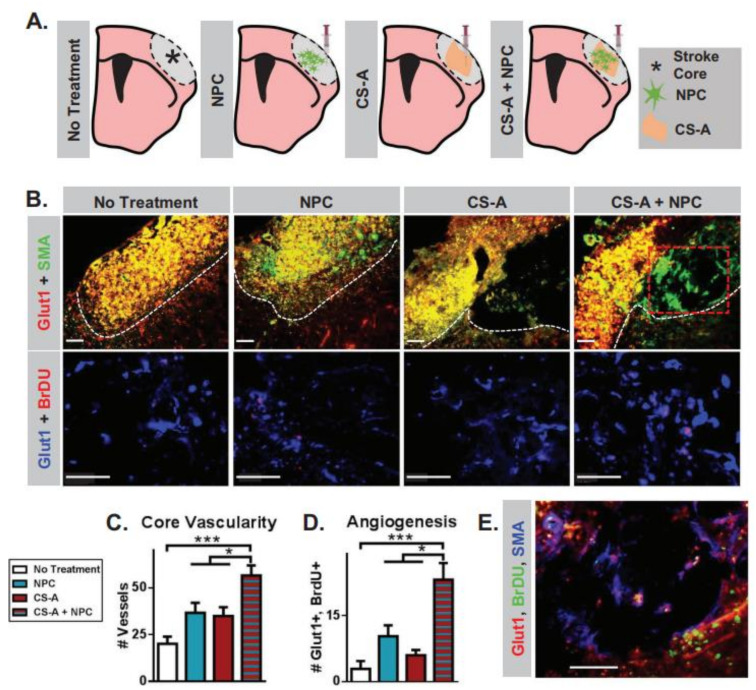
(**A**) Schematic illustration of treatment groups following strokes in mice. (**B**) Vessels in the necrotic stroke core across treatments. (**C**) Newly formed endothelial cells across treatments. (**D**) Analysis of the vascularity angiogenesis. (**E**) Muscular artery formation images. *, *** indicate *p* < 0.05, *p* < 0.0005, respectively (1-way ANOVA, Tukey’s post-hoc). Reproduced with permission from [65]. Copyright 2020, Wiley-VCH.

Although HA shows advantageous extracellular matrix properties for tissue repair applications, HA lacks the functional elements that are essential for increasing the retention of trophic factors. Some other ECM glycosaminoglycans contain functional elements that allow for endogenously regulated trophic signaling, thus enhancing regeneration after a stroke. For example, chondroitin sulfate (CS), a common component in cell surfaces and the extracellular matrix, usually functions as a multifunctional sulfated glycosaminoglycan that is involved in regeneration in the ischemic brain. The CS disaccharide chains contain sulfated elements that are attached to native brain CS proteoglycans and play a role in growth factor retention [59,60,61]. CS-A is involved in endogenous neural stem cell differentiation and migration. Previous studies also found that CS-A could support in vitro NSCs and in vivo transplanted cells after brain injury [62,63,64]. CS-A hydrogels were therefore chosen to serve as effective vehicles for NSC-mediated repair in ischemic strokes. As shown in Figure 4, CS-A loaded with NPCs improved angiogenesis in the brain. In the treatment of ischemic strokes, the encapsulation of neural stem cells within a CS-A hydrogel could enhance transplantation for stroke and the brain recovery capabilities of NPCs [65]. Chitosan’s application potential in stroke treatment was also reported previously. For example, an MSC-loaded chitosan–collagen hydrogel was used for stroke treatment [66]. The ability of chitosan-based hydrogels to improve neural neurodegenerative therapy was proven by encapsulating progenitor cells within the hydrogels [67,68]. In another work, an MSC-loaded chitosan-based thermosensitive composite hydrogel was found to reduce cell death, induce neurotrophic factor secretion and improve the survival of endogenous neural cells in the injured brain [69]. A hyaluronan/collagen/heparin hydrogel matrix that could be injected was used to create a favorable environment for stem/progenitor transplantation into the infarct cavity in strokes. This hydrogel contains the following components: hyaluronan (which is well known as a major component of the brain extracellular matrix), collagen (promotes cell attachment) and heparin (binds and stabilizes growth factors and functions as an important constituent in neuronal differentiation for NPCs) [70]. ECM hydrogel implantation offers a new therapeutic platform for stroke treatment by inducing brain tissue regeneration within the injured cavity. The therapeutic effectiveness of ECM hydrogels should be evaluated in the evolving postinjury environment. Corina Damian et al. studied how the time points at which ECM hydrogels were implanted poststroke influenced their delivery, degradation, host response and regeneration. They found that the therapeutic window is dependent on a tissue cavity that allowed the bio-scaffold to be delivered. An optimal therapeutic time window for bio-scaffold-triggered regeneration of brain tissue and recovery occurred between 14 days and 28 days poststroke [71]. Silk has been used in humans for a long time [72]. It has been approved for clinical application as a suture and surgical mesh [73]. The excellent mechanical properties, biodegradability and biocompatibility of silk make it an ideal candidate for wider applications than those clinically approved at this time [74]. Notably, silk fibers could be prepared into an aqueous silk solution, which could be further processed into cross-linked hydrogels [75]. Silk can also be triggered to self-assemble and form a hydrogel. Silk hydrogels have been widely researched in preclinical studies as carriers of drugs and cells, especially in chronic stroke therapy [76,77]. Silk fibroin hydrogels exhibit several advantages, including low cell-binding properties, tunable physiological brain requirements and non-swelling behavior, supporting the growth and differentiation of stem cell-based payloads, such as pluripotent and MSC types [78,79]. The biocompatibility of silk fibroin hydrogels was confirmed in the absence of any payload in both normal and MCAO mice. Almost no adverse reactions occurred in these studies [80,81]. Self-assembled silk hydrogels were studied as an MSC support matrix to explore cell therapy in minimally invasive brain applications. To produce a delivery system with appropriate physical and chemical properties, such as controllable solution–gel kinetics, a self-assembled silk hydrogel was fabricated as a platform with optimum MSC hydrogel matrix conditions for brain repair [82]. Sericin protein is naturally derived with cell-adhesive, neurotrophic and neuroprotective properties and can be cross-linked to prepare an injectable hydrogel, which is usable as a carrier to deliver neurons for tissue repair applications [83]. In addition, a novel cross-linked sericin hydrogel was prepared as an in vivo effective cell delivery system for brain repair. When transplanted in vivo, the cross-linked sericin hydrogel showed good biosafety and effectively promoted cell proliferation [84]. 

### 2.3. Peptide-Based Hydrogels

Many synthetic and naturally derived hydrogels have been applied in ischemic stroke therapy and brain tissue recovery. However, these biomaterials have some undesired properties that limit their applications. For example, as widely applied hydrogels, HAMC and alginate hydrogels are nearly non-adhesive to various cells, while for another kind of commonly used biomaterial, PLA/PLGA, their degraded products change the pH in the microenvironment of host tissues. Therefore, in addition to intrinsic neurotrophic activity, biomaterials are desired to be adhesive to cells, and their degraded substances should be neuronally biocompatible without inducing side effects on tissues. In recent years, self-assembling peptide sequences have been designed to form hydrogels [85]. This type of self-assembling peptide hydrogel exhibits a great advantage because they are ECM-like biomimetic three-dimensional structures, and their degraded products are amino acids with good biocompatibility [86,87]. Furthermore, the sol–gel transition occurs under physiological conditions so that the hydrogel could be well suited to lesion cavities after in situ injection [88,89]. Parish, C.L. et al. developed a novel spontaneous self-assembling peptide hydrogel and proved that this hydrogel was able to support neural progenitor grafts in the injured brain after a stroke. [90]. However, the low mechanical problem of self-assembled peptides (SAPs) limits their application, which is mainly caused by weak noncovalent bond interactions. A method that could avoid chemical cross-linking and UV irradiation was proposed to enhance the mechanical properties of the peptide hydrogels, and hydrogels were formed from a SAP solution in situ by a self-assembly process under physiological conditions [91].

The earliest designed and most widely used SAPs usually comprise repeated amino acid residues that are alternatively hydrophilic and hydrophobic. EAK16, as one of these kinds of SAPs, was first reported by Zhang et al. in 1993 [92]. EAK16 contains the amino acid sequence Ac-(AEAEAKAK)2-CONH2, which can self-assemble into β-sheets. The EAK16 sequence could be further modified by changing some amino acids to form the well-known RADA16 (Ac-(RARADADA)2-CONH2) [93]. RADA-like hydrogels were first explored for application in brain injury repair by Ellis-Behnke et al. in 2006. The authors injected RADA16 into an injured site in the midbrain of hamsters. The results showed that RADA16 facilitated axonal growth within the tissue gap [94]. RADA16 was commercialized with the commercial name PuraMatrix in 2002.

Later, researchers successfully used RADA16 for stem cell encapsulation by functionalizing this peptide with cell-adhesive ligands. These functional domains extend away from RADA16 and do not disrupt its self-assembly property. The attachment of bone marrow homing peptides to RADA16 improved the survival of encapsulated NSCs without the addition of any other therapeutic growth factors or neurotrophic factors [95]. Similarly, RADA16-IKVAV SAP hydrogels were also proven to increase the survival of loaded NSCs and decrease glial astrocyte formation [96]. Furthermore, after modification, RADA16 hydrogels could become angiogenic. For example, RADA16 modified with SAP PRG and KLT improved endothelial cell sprouting and vessel formation [97]. Therefore, angiogenic hydrogels may show great potential to promote the endogenous repair of injured penumbra in strokes. In another work, a self-assembling hydrogel based on the peptide IKVAV was shown to support poststroke survival and maturation of cortical neurons implanted into the ischemic area [98]. These studies demonstrated that there is a relationship between cells and ECM molecules within hydrogels that has an important effect on cell fate. The existence of ECM molecules facilitating cell adhesion and growth should be well considered when designing hydrogels for delivering cells to brain tissue. In addition, the peptide sequences that are capable of self-assembling and forming scaffolds to facilitate NSC attachment should be well investigated.

**Figure 5:**
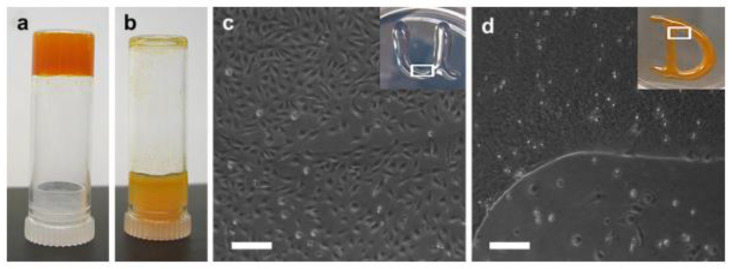
(**a**,**b**) Characterization of MAX8 hydrogels loaded with curcumin. (**c**,**d**) DAOY cells seeded with MAX8 hydrogels with or without curcumin. Reproduced with permission from [103]. Copyright 2011, Elsevier.

Due to their tunable stiffness and the ability to protect containers from degradation, SAP hydrogels could also be used to control the local release of payloads in the stroke infarct [99]. For example, an injectable hydrogel named RADA16 was created from SAP sequences, and the obtained hydrogel showed an ability to maintain a high level of water content to release different protein products. RADA16 was thus applied to deliver both VEGF and Ang1 to encourage vascular regeneration and neuronal plasticity in brain tissue. The author demonstrated that the intralesional injection of this in situ-forming hydrogel composite can promote long-term brain regeneration and enhance the recovery of brain function after a stroke in the chronic phase [100]. In another study, the author tuned the peptide sequence to retain the assembly property, and the functional epitope was observed at a high density on the surface of nanofibrils with the resultant Fmoc–DDIKVAV. The epitope peptide sequence encoded the binding domain of laminin, and two aspartate residues were added to the sequence to enable the self-assembling property of this scaffold. The release profile of BDNF from the tissue-specific peptide hydrogel was then studied. An investigation in a rat model showed that BDNF-loaded scaffolds could promote the survival of neural progenitors. Therefore, delivering therapeutic proteins with injectable hydrogel systems could significantly enhance neuroprotection [101]. MAX8 has been widely studied as a carrier of a variety of neurotrophic factors, such as BDNF. For instance, it was found that NGF and BDNF could be released sustainably when encapsulated within MAX8 for half a month. Interestingly, the release rate of these factors could be altered by adjusting the hydrogel concentration. The higher concentrations led to slower release because of the decreased porosity [102]. By coincubation of MAX8 hydrogel systems with PC12 cells, the authors proved that NGF and BDNF released from the hydrogel retained their activity well and prolonged their half-life. In addition, the physiochemical properties of the hydrogel remained unchanged. Apart from growth factor delivery, small-molecular drugs, such as curcumin, have also been reported to be loaded within MAX8 hydrogels, and the hydrogel system is shown in Figure 5 [103]. These findings suggest that the MAX8 system is a potential platform in ischemic stroke therapy by delivering various therapeutic drugs into the stroke cavity for sustained release.

Moreover, synthetic hydrogels, not so widely, are also applied in ischemic stroke therapy. As mentioned before, an HA–PLGA hydrogel was prepared and applied for neural regeneration after strokes [52]. A hydrogel based on pluronic–chitosan and aniline–pentamer was prepared and loaded with VEGF to improve the recovery of ischemia imperfection in the hippocampus [104]. During the preparation of the hydrogel, pluronic gives the injectability property to the hydrogel, while CS has ECM-like properties. Poly (trimeth ylene carbonate)_15_-F127-poly (trimethylene carbonate)_15_ (PTMC_15_-F127-PTMC_15_, PFP) is another synthesized thermo-sensitive hydrogel with good biocompatibility. Neural stem cells (NSCs) and neurotrophic factors, including brain-derived neurotrophic factor, neurotrophin-3 and nerve growth factor, were collectively loaded into a PFP polymer hydrogel for controlled release. Afterwards, the NSC polymer scaffold was implanted in rat brains of MCAO mice. The results verified that the PFP scaffold could release BDNF, NGF and NT-3 sustainably to support the differentiation of seeded NSCs [105].

## 3. Conclusions

As an acute disease, strokes have become the most common cause of disability worldwide, and both early diagnosis and timely therapies are necessary to treat stroke. However, despite widespread clinical practice and reperfusion therapies, most survivors experience a high recurrence rate and neurological impairment, which has a severe influence on their quality of life and becomes a heavy burden on society. Novel treatment methods to reduce cellular death and enhance endogenous brain tissue repair may improve stroke outcomes. However, despite promising results in experimental stroke models, most of the therapies failed during their translation to clinical trials. The reason for failure may be the short half-life or severe side effects of therapeutic drugs and the low survival rate of transplanted cells for cell-based therapy. Therefore, the lack of a successful clinical therapy that could effectively enhance long-term brain tissue recovery has become a heavy clinical and social burden, and new therapy strategies urgently need to be developed. The targeted delivery of drugs to the injured brain is necessary for the effective treatment of ischemic strokes. Although various therapeutic formulations have been developed, the basic problem of how they reach ischemic stroke lesions specifically remains unaddressed. The BBB is an important hurdle for systemically administered therapeutics, which is why systemic therapy methods try to target the leaky vasculature of the brain and ultimately cause brain failure. Therefore, a local delivery strategy represents a promising method that can completely bypass the BBB. This strategy is advantageous because it never causes off-target events and reduces the drug dose needed to reach therapeutic effects in stroke infarctions. In addition, systemic exposure and side effects are eliminated, and the possibility of degradation is decreased.

During the past decades, the application of biomaterials for the local release of therapeutics has been widely reported. Recent improvements in tissue engineering have led to the development of injectable hydrogels that could serve as carriers for various agents. Utilizing hydrogels is advantageous because they promote repair processes due to their ability to deliver payloads sustainably and controllably, offering mechanical support and physical filling to the host tissue. In addition, hydrogel-based delivery systems could achieve combination therapies by co-incorporating therapeutics. Since strokes involve multiple mechanisms and a complicated pathophysiology, combination therapies are more likely to achieve a significant result. For example, combined drug and cell delivery represents a promising strategy that efficiently enhances tissue recovery and regeneration. Other combination therapies, such as the sequential release of different drugs with environmental triggers, are expected to significantly enhance neural recovery and regeneration.

In summary, this review introduced the pathological characteristics of strokes, the current therapy methods and recent advances in the development of hydrogels to treat strokes. We have discussed various hydrogel-based platforms that have been employed in ischemic stroke therapy. It is necessary to further understand the pathogenesis of ischemic strokes and the interaction of the hydrogel system with brain tissues. The hydrogels discussed in this review show great potential for brain tissue regeneration and offer a unique platform to develop novel ways to improve the therapeutic effects for ischemic strokes.

## Abbreviations

CNS	central nervous system
AIS	Acute ischemic stroke
DAMPs	Damage-associated molecular patterns
BBB	Blood–brain barrier
BDNF	Brain-derived neurotrophic factor
rt-PA	Recombinant tissue plasminogen activator
FDA	Food and Drug Administration
HA	Hyaluronan
MC	Methylcellulose
MSC	Mesenchymal stem cell
cNEPs	Cortically specified neuroepithelial progenitor cells
NSPC	neural stem/progenitor cell
NSC	Neural stem cell
VEGF	Vascular endothelial growth factor
PLGA	poly (lactic-co-glycolic acid)
PLL	poly-L-lysine
MCAO	Middle cerebral artery occlusion
EGF	Epidermal Growth Factor
EPO	Erythropoietin
BIO	6-Bromoindirubin-3′-oxime
ECM	Extracellular matrix
CS	chondroitin sulfate
SAPs	self-assembled peptides
